# News and Miscellany

**Published:** 1903-05

**Authors:** 


### News and Miscellany.

Too bad that the breezy and brilliant I. N. Love disappointed us. Everybody was disappointed. I'll never speak to him again.

Dr. J. N. Musser, of Philadelphia, was elected President American Medical Association at the New Orleans meeting; Simmons as Secretary, and Newman as Treasurer, holdover.

I was glad to make the acquaintance of Dr. J. N. McCormack, of Bowling Green, Ky. He "organized" us at San Antonio. He is a delightful fellow, genial and jolly, as are all true Scotchmen.

Will Repair Your Electric Machines.—Static and all Electra Medical Apparatus put in running order. I am also agent for electrical and X-ray apparatus. Oliver Brush, 710 Colorado Street, Austin, Texas.

Examinations of urine, sputum, blood, pathological specimens, etc., made at reasonable rates by New Orleans Clinical Laboratory, 124 Barrone Street, New Orleans. O. L. Pothier, M. D., Charity Hospital. I. I. Lemann, M. D., Secretary. J. B. Guthrie, M. D.

That "barbecue" at Hot Wells was a sausage and beer affair. It was delightful, nevertheless. A fellow sitting by me said: "I never sausage a supper," and then had the audacity to ask if I would oblige him bolognaing him a dollar. I killed him; brained him on the spot.

Dr. Thomas B. Grayson, of Winkler, Texas, died recently at his home in that place, aged 70 years. He was born March 1,

1833; graduated at Tulane University, New Orleans, La., in 1855; began practice at the age of 22; was surgeon in Company B, Second Battalion, Waul's Legion; was stationed at Galveston, Texas, 1864 and 1865.

---

SLEEPING-CAR DISINFECTION.—The State Board of Health of Louisiana has passed a resolution requiring every sleeping-car company operating in Louisiana to thoroughly disinfect each sleeping-car after every round trip, under such regulations as the board may prescribe; provided, that after personal investigation the quarantine committee finds such methods of disinfection necessary.

---

THE Eastern office of the Abbott Alkaloidal Company is located ot 50 West Broadway, New York; formerly 93 Broad Street. Their Eastern business has increased so rapidly within the last year, under the management of Mr. N. B. Harris, that large and more commodious quarters were necessary. Friends will receive a hearty reception from Mr. Harris at any time in the new home.

SEEING IS BELIEVING.—State Health Officer Tabor, together with a representative each of the Louisiana and Alabama Boards, has gone to Cuba, to personally look into the sanitary condition of the island. These gentlemen believe that one eye-sight is worth ten hearsays. They will govern their action relative to ceasing disinfection of vessels from Cuban ports, as suggested by the Marine Hospital Service, according to what they see. They do their own thinking.

---

THE STATE BOARD OF MEDICAL EXAMINERS FOR TEXAS.—The Governor re-appointed the old board, with the exception of Dr. Paschal, elected President State Medical Association, and not an applicant for re-appointment. Dr. S. T. Turner, of El Paso, was appointed in his stead. The members are: Wilson, of Sherman; Smith, of Austin; Scott, of Houston; Reuss, of Cuero: Jones, of

Gonzales; Burroughs, of Buffalo; Evans, of Palestine; Jenkins, of Daingerfield, and Turner, of El Paso.

---

THE NEW CONSTITUTION adopted by State Medical Association at San Antonio, will be printed in pamphlet form; 5,000 copies, and copies will be sent to every county and district society in the State for distribution. County societies or physicians intending to organize county societies, can get, free of charge, copies, as many as they need, of the constitution proposed for county societies, by writing to Dr. Geo. H. Simmons, Secretary of the American Medical Association, and editor of the *Journal American Medical Association,* at Chicago.

---

THE STRANGE CASE OF DR. BRUNO.—I've written another book, and that's what I call it. There's not a joke in it. It is a tragedy. It is strenuous. It is a problem in the chemistry of physiology or something like, around which is woven a romance of a very unique and original kind. It is a very remarkable story. It will be run serially through the "Red Back," beginning with Vol. XIX, No. 1 (July), and then a small edition will be issued in book form. If you want a copy, subscribe for it now, and send me a dollar. It will be sold only in this way, and there will be only 500. It will run through in five issues.

---

LOUISIANA KEEPS HER OWN QUARANTINE.—The State Board of Health has decided not to make its quarantine regulations conform with those of United States Public Health and Marine Hospital Service, or to use the marine hospital inspectors instead of its own, or to relieve coastwise vessels of the necessity of inspection on entering New Orleans. These changes were suggested by Dr. Lindsley, of Havana. The Louisiana board will maintain its own inspectors at all gulf ports where there is considered to be any danger of the importation of disease.—*Journal American Medical Association.*

(Texas will keep her own, also, I think.)

---

ASYLUM CHANGES.—Dr. Frank Ross, Second Assistant Physician State Insane Asylum, Austin, has resigned. Dr. John H. Foster, Third Assistant, has been promoted to fill the vacancy, and Dr. Louis Kirk, of Austin, has been appointed Third Assistant. Dr. G. H. Moody, First Assistant Physician Southwest Texas Insane Asylum, San Antonio, has also resigned. Drs. Ross and Moody will go to Europe to study. Dr. W. L. Allison, Second

Assistant at the Southwest Texas Insane Asylum, has been promoted to fill the vacancy caused by Dr. Moody's resignation, and Dr. James Greenwood, of Seymour, Texas, has been appointed Second Assistant Physician, vice Allison, moved up a notch.

---

You should just have seen Old King dancing the cake-walk with a pretty widow. You would have died laughing. Everybody knows the dear, delightful old fellow. Hadra said at the banquet, last October, when King sang about his gathering shells at the seabeat shore—as he has done at every banquet for twenty or thirty years—that he didn't believe it; didn't believe King would know a shell if he met it in the road. Growing reminiscent, Hadra said: "The first time I ever heard King sing that song he was traveling with a Wizard Oil wagon." "Hold on there, Hadra," said King, "everybody present may not know what a d—n l—r you are." As James Whitcomb Riley says: "You'd oughter heard 'em yell."

---

My esteemed friend, Dr. R. W. Skipper, of Lovelady, writes as follows of the loss of his office and complete file of the "Red Back," from Vol. 1, No. 1, to date:

Lovelady, Texas, April 29, 1903.

*Dr. F. E. Daniel, Austin, Texas.*

Dear Doctor: On last Saturday night, the 25th inst., I lost my office, together with a part of its contents, by fire. I had one of the most complete files of the Journal that could be found; not a missing number of the "Red Back" since its first issue, and today I can only find six copies of that invaluable publication. Three of those were at my residence and three were carried out with what books were saved.

Fraternally yours,

R. W. Skipper.

---

State Board of Medical Examiners—An object lesson for a Senator.—The State Board of Medical Examiners (regular) met at Austin, April 20th to 24th. They examined ninety-five applicants, most of them graduates of the Medical Department, University of Texas. These young fellows stood splendid examinations, and all got their license. There were ten who failed; most of them, I learn, were from other States. During the examinations, which took place on the ground floor of the Driskill Hotel, in a large room separated from the main entrance by glass partitions, a well known member of the late State Senate asked me:

"What is going on in there, doctor?" I said: "Come here, Senator; this is an object lesson for you. I wish every Senator and member could see it. It is the process of examining, by the State Board, nearly a hundred of the recent graduates of our University Medical College. They are educated free by the State. They are required to stand a strict examination for matriculating. After four years study, and a laboratory course, they are required to stand a thorough examination for the degree. *Then* the State requires this, in addition, before they are licensed to practice. You gentlemen exempt from all restrictions the 'drugless ones;' men educated nowhere, and graduates in nothing! Is that right, Senator? It is simply *idiotic.*" He was converted on the spot.

